# A Review of Hypertension and Diabetes Protocols for Medical Service Trips (MSTs) in Latin America and the Caribbean

**DOI:** 10.29024/aogh.2387

**Published:** 2018-11-05

**Authors:** Christopher Dainton, Jenifer Truong, Charlene H. Chu

**Affiliations:** 1 McMaster University, CA; 2 Grand River Hospital, Kitchener, CA; 3 Medical Service Trip.com, CA; 4 Toronto Rehabilitation Institute-University Health Network, CA

## Abstract

**Background:**

Hypertension and diabetes are among the most common chronic conditions that may be managed on short-term, primary care medical service trips (MSTs) in Latin America and the Caribbean (LAC), but the quality of patient care delivered remains unclear.

**Objective:**

This study summarizes protocols that Western volunteer clinicians use in managing these patients, and highlights their commonalities, differences, and potential limitations.

**Methods:**

A systematic web search was used to identify organizations operating MSTs in LAC. Organizations were contacted by email or through their websites to obtain clinical protocols intended for use on their brigades. These protocols were qualitatively analyzed, and recommendations were categorized into clinical assessment, non-pharmacologic recommendations, and pharmacologic recommendations.

**Findings:**

Two hundred twenty-five organizations were identified and contacted, and protocols were obtained for 20 of these. Eleven (55%) of these protocols discussed hypertension, and 10 (50%) discussed diabetes. Only one protocol provided any literature support for its recommendations.

**Conclusions:**

The analyzed protocols may give insight into context-specific realities of practice on MSTs, but they often neglected key aspects of clinical management that are emphasized in international guidelines. This study is an initial step in clinical guidelines development for MSTs operating in LAC.

## Introduction

Short-term, primary care medical service trips (MSTs) to low- and middle-income countries (LMICs) are increasingly accessible to clinicians and trainees. Otherwise known as “medical missions” or “brigades”, these activities often involve mobile clinics with limited resources providing basic primary care services to remote communities [1]. Based on an epidemiologic study of five Latin American MST locations, hypertension (1.6–7.9% prevalence) and diabetes (0.3–4% prevalence) are among the most common chronic conditions that may be managed on brigades [2].

Clinical guidelines are traditionally used to make care more evidence-based and consistent [3]. They would be valuable in supporting Western clinicians who encounter unique practice challenges on MSTs, such as scarcity of resources, limited health literacy of patients, and unclear clinical follow-up. A recent integrative review, however, demonstrated evidence of a lack of published clinical guidelines and protocols for practice in MST settings in Latin America and the Caribbean [4].

Despite these review findings, many non-government organizations (NGOs) possess *unpublished* training protocols that are distributed to the clinicians who travel with them. It is important to understand the content of these protocols, as well as the process used to develop them, as one element of describing quality of care on MSTs. This is particularly relevant to chronic conditions such as hypertension and diabetes, that may have profound macro and microvascular sequelae.

To our knowledge, no attempt has been made to collect, summarize, and consolidate these unpublished clinical protocols. The following study addresses the gap in MST guidelines development by summarizing and highlighting the commonalities, differences, and potential limitations of unpublished hypertension and diabetes protocols used by primary care MSTs in Latin America and the Caribbean.

## Methods

This descriptive study forms part of a larger initiative aimed at locating and describing unpublished clinical protocols utilized by MSTs in Latin America and the Caribbean. Clinical protocols related to general pain, gastrointestinal symptoms, respiratory symptoms, gynecologic symptoms, urinary symptoms, dermatologic conditions, hypertension, and diabetes were collected; however, this paper presents only the findings related to hypertension and diabetes protocols.

### Sampling strategy

NGOs currently operating short-term, primary care MSTs in Latin America or the Caribbean were sampled and identified in three ways. First, several online databases were used (www.missionfinder.org, www.medicalmissions.org, www.mmex.org, www.globalhealth.arizona.edu, www.internationalhealthvolunteers.org) to identify NGOs. Second, a systematic Google web search was conducted using combinations of the terms: “medical missions”, “short term missions”, and “medical mission organizations”, combined with each country in Latin America and the Caribbean [5]. Based on a similar search for short-term MSTs conducted by Lasker [6], the Google search was extended to include different combinations of the terms: “international health volunteering”, “Christian health volunteering”, “religious health volunteering”, “corporate global health volunteering”, “international health fellowships”, “international health educational opportunities”, “global health director”, “international service learning”, “global health elective”, “medical school international internships”, “intercultural learning”, “global health volunteer projects university”, and “international volunteer organizations”. Third, organizations were also located through social media, using the Twitter hashtags “medical mission” and “global health”. These searches were performed every two months between April 17, 2014 and July 20, 2015 to find and include as many organizations as possible due to the diversity of web presence and constantly changing NGO landscape.

Organizations were contacted by the research team if they: facilitated North American clinicians (physician, physician assistant, osteopath, or nurse practitioner) traveling to Latin America or the Caribbean, and had operated at least one short-term (i.e. less than one month) primary care MST in the previous year. Exclusion criteria were organizations that exclusively performed specialty or surgical trips, as well as trips that did not involve direct patient care by North American clinicians. Organizations with multiple chapters (i.e. university MST organizations) were treated as a single unified parent organization and only one chapter was contacted.

### Data collection

#### Procedures

We obtained the following data from the website of each eligible organization and entered it into an Excel spreadsheet: its base of operations, countries served, frequency of MSTs to Latin America, clinical setting of the MST (rural, urban), number and type of providers, diagnostic resources available during the MST, and whether the organization was faith-based or secular.

We searched each NGO website and downloaded any medical provider handbook, clinical protocols document, or description of clinical management on an MST. If no such documents could be found, an attempt was made to contact the NGO directly by the email address posted on the website. If NGOs were contacted by email, a prepared email explained the study purpose and then asked: “Do you have any specific clinical protocols or training documents for clinicians working in low resource mission settings?” We documented the number of NGOs contacted, and any reasons provided for the absence of protocols or for declining to share protocols. All clinical protocols received were saved on a secure cloud drive.

#### Analysis

An Excel spreadsheet was used to systematically classify and code data from the received protocols into categories related to clinical assessment, non-pharmacologic management suggestions, and pharmacologic management suggestions. The most common protocol statements in each category were identified, organized thematically, and checked for the inclusion of supporting references.

## Results

**Figure 1:**
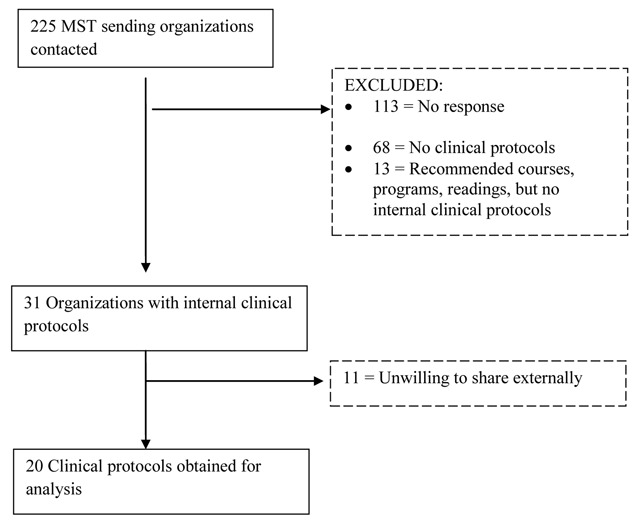
Flow chart of NGOs operating short-term primary care MSTs that were contacted to share clinical protocols.

Figure 1 indicates the number of protocols retrieved and the responses of NGOs to information requests. The search strategy generated 225 unique NGOs. Of these NGOs, 113 (50.2%) did not respond to our attempts to contact them. Sixty-eight (30.2%) responded that they did not use any clinical protocols, while 13 (5.8%) responded that they had recommended pre-departure readings but no specific protocols. Although structured analysis of the reasons for lacking protocols was outside the scope of this study, organizations without protocols responded that treatment offered was “the same as in the US”, “on the spot medicine”, “mostly basic healthcare issues”, “mostly basic family medicine”, and described the presence of “very few diagnostic syndromes, and they are all treated the same out of necessity”.

Thirty-one NGOs (13.8%) used clinical protocols and 20 (64%) of these were obtained and included in this analysis (Table 1). Of the 20 organizations for whom protocols were obtained, only two served in South America, and all remaining groups served in Central America and the Caribbean. The most common locations were Haiti (35%, n = 7), Honduras (25%, n = 5), Guatemala (25%, n = 5), and Nicaragua (20%, n = 4). Six organizations served in more than one country. All organizations except one operated mobile or standing clinics in rural locations, and the remaining organization operated in an urban setting. Six organizations were faith based, while the other fourteen were secular. The number of MSTs that each organization operated annually was highly variable, ranging from one to hundreds.

**Table 1:** Characteristics of NGOs providing clinical protocols for short-term, primary care MSTs in Latin America and the Caribbean.

MST ID	Locations served	Trips per year	Format and setting	Faith-based or secular

1	Nicaragua	4	Rural mobile brigades and standing clinic	Secular
2	Guatemala	Variable	Rural clinics in two villages	Secular
3	Haiti	Variable	Urban clinic in Cite Soleil	Faith-based
4	Dominican Republic	3	Rural clinic in Puerto Plata province	Secular
5	Haiti	7	Rural	Secular
6	Honduras, Ecuador, Belize, Guyana, Guatemala	5	Rural and urban mobile brigades	Faith-based
7	Ecuador, Guatemala, Dominican Republic	~100 (spread over 6 sites)	Rural and urban mobile brigades at two sites	Secular
8	Ecuador	3	Rural mobile brigades in schools or clinics in health centres	Secular
9	Honduras	1	Rural mobile brigades in 20 villages	Faith-based
10	Jamaica, Haiti	4	Rural and urban mobile brigades, as well as some permanent clinics/hospital medicine	Secular
11	Haiti	4	Rural clinic	Secular
12	Guatemala, Nicaragua	3	Rural mobile clinics	Faith-based
13	Nicaragua, Honduras	51	Rural mobile brigades, as well as hospital-based	Faith-based
14	Guatemala	12	Rural hospital	Secular
15	Honduras	Up to 50	Rural mobile brigades in community schools and churches	Faith-based
16	Haiti	Variable	Urban and rural mobile brigades and clinics	Secular
17	Dominican Republic, Haiti	12 to 15	Rural mobile clinics in bateys	Secular
18	Honduras, Nicaragua, Panama	“Hundreds”	Rural mobile clinics in community centres or schools	Secular
19	Honduras	4	Rural mobile brigades as well as specialty health services	Secular
20	Haiti	~40	Rural mobile clinics in 14 villages (Western operated) and standing clinics (Haitian operated)	Faith-based

### Hypertension clinical protocols

**Figure 2:**
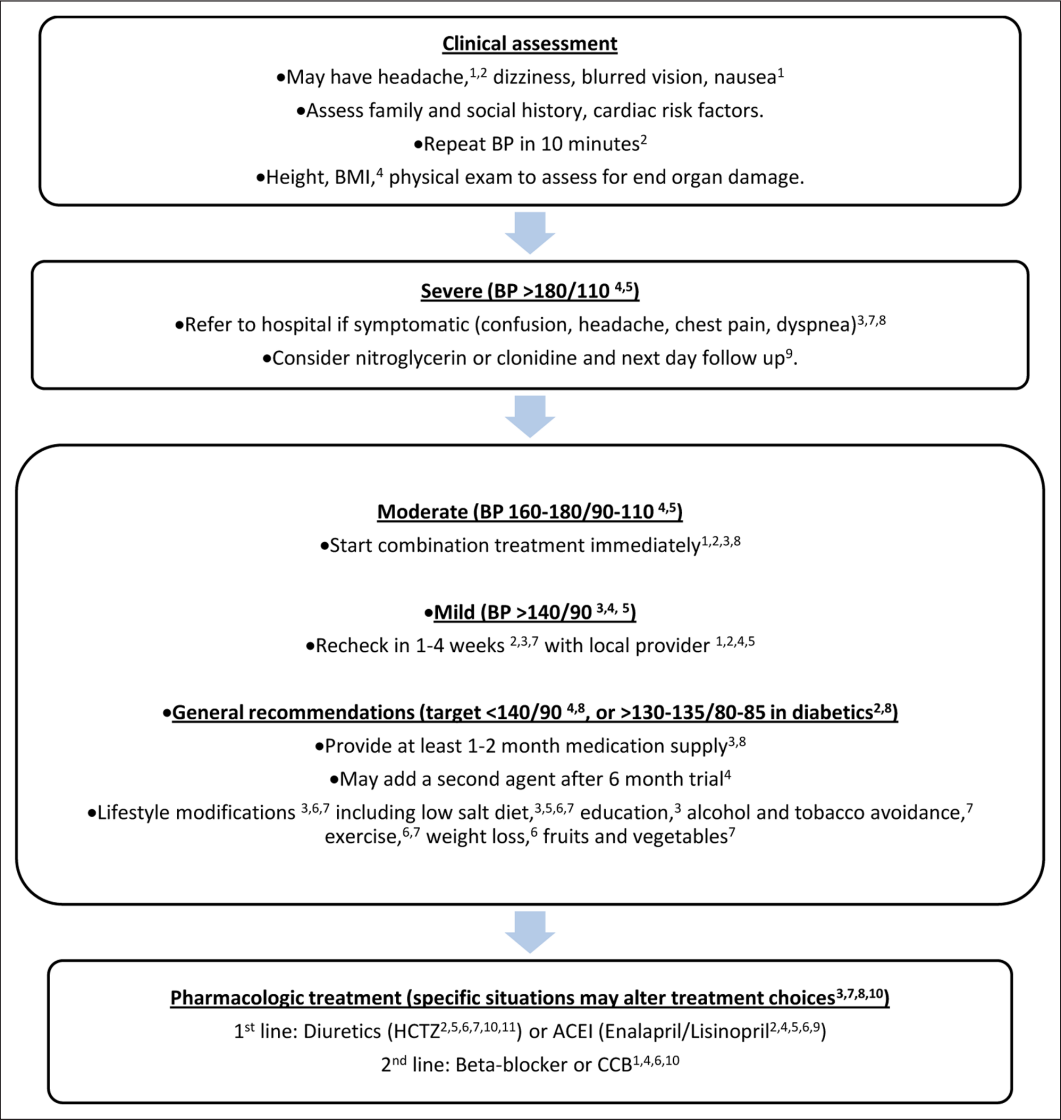
Most common recommendations for management of hypertension in short-term, primary care MST settings in Latin American and Caribbean, compiled from a composite of protocols from 20 NGOs.

Of the 20 protocols obtained, 11 discussed hypertension (55%; Organizations 1–11), and the most common recommendations are summarized in Figure 2. Only one guideline (Organization 7) provided literature support for its management suggestions, citing the 2003 WHO/ISH Hypertension Guidelines [7].

Seven protocols defined hypertension as a blood pressure measurement >140/90 (Organizations 2–8) while the others did not provide any parameters. With respect to clinical assessment, four organizations (1–4) mentioned that patients with hypertension may be asymptomatic or may present with non-specific symptoms such as headache, dizziness, blurred vision, and nausea. The most commonly recommended non-pharmacological intervention was lifestyle modification (Organization 2–8), which included limiting salt, alcohol, and tobacco, increasing exercise, and eating a healthy diet. Nine of the 11 organizations (Organizations 1, 2, 4, 5, 6, 8–11) suggested diuretics or an ACE inhibitor as first-line therapy and a calcium channel blocker (CCB) as second-line therapy. One NGO (Organization 2) recommended that pharmacologic therapy be reserved for patients with elevated blood pressure readings on two separate clinic visits. Only four protocols (Organization 1–3, 5) explicitly discussed clinical follow-up within two to four weeks. Four protocols (Organization 2, 4, 7, 8) included additional recommendations for severe hypertension or hypertensive emergencies, which included the addition of another antihypertensive drug and referral to a medical center.

### Diabetes

**Figure 3:**
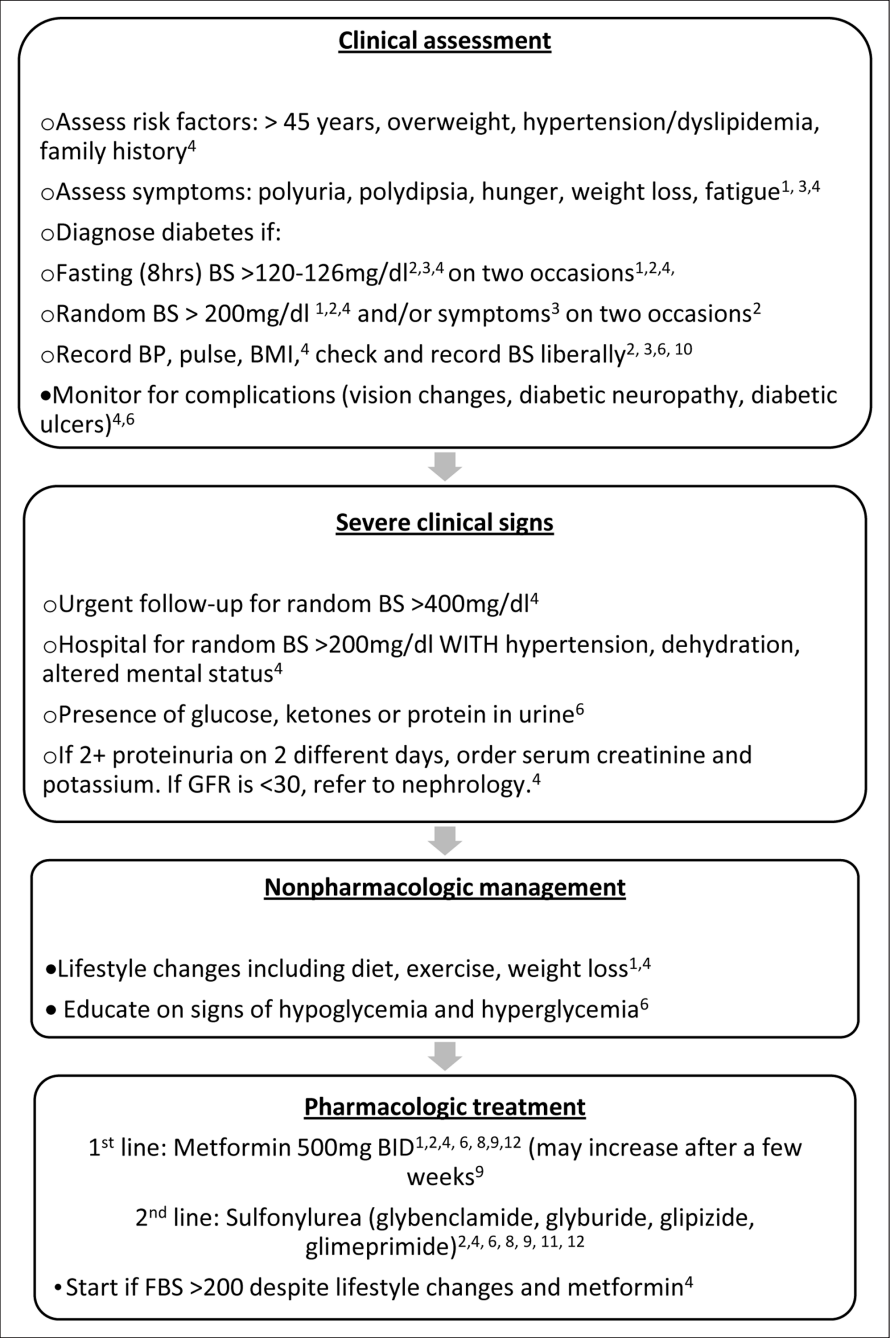
Most common recommendations for management of diabetes in short-term, primary MST settings in Latin America and the Caribbean, compiled from a composite of protocols from 20 NGOs.

Of the 20 protocols obtained, 10 discussed diabetes (50%; Organization 1–4, 6, 8, 9–12), and the most common recommendations are summarized in Figure 3. Four of the 10 organizations with diabetes protocols (Organization 1–4) suggested diagnosis based on a fasting blood glucose ≥126 mg/dl or plasma glucose of ≥200 mg/dl. Organizations 1, 4, and 6 suggested assessment on history and physical examination for indications of micro- and macrovascular damage. All 10 protocols (Organization 1–4, 6, 8, 9–12) recommended metformin as first-line pharmacologic therapy and a sulfonylurea as second-line therapy. No protocols specifically discussed diagnosis and management of Type I diabetes.

## Discussion

This review is the first to consolidate unpublished protocols for commonly treated chronic conditions on MSTs operating in Latin America. Eleven of the 20 organizations that had protocols included recommendations for managing either hypertension or diabetes, suggesting that these conditions are recognized as relevant to such groups. None of the protocols, however, were the product of rigorous literature searches, and most were not backed by any literature citations. Therefore, it is unclear whether such protocols are based on robust evidence, availability of scarce resources, or simply expert opinion. This review gives an indication of the current landscape of medical management on MSTs in austere settings, but limited insight into whether treatments are efficient, effective, sustainable, or cost-effective. In the following paragraphs, we discuss the hypertension and diabetes protocols in the context of international practice guidelines to identify the strengths and weaknesses of current MST practice recommendations.

### Hypertension

The six protocols with objective definitions for hypertension were in substantial concordance with International Society of Hypertension (ISH) recommendations that stratify the diagnosis as either mild, moderate, or severe [7]. However, while most international recommendations also draw attention to concerning signs and symptoms of hypertensive emergency that warrant a higher level of care, only five protocols discussed such features. A clear case definition for those requiring tertiary care referral would be particularly valuable for MST clinicians working in remote settings, where the financial and logistic cost of emergency transport presents a barrier to both patients and NGOs.

Lifestyle changes were recommended by the MST protocols with varying degrees of comprehensiveness, but generally mirrored World Health Organization (WHO) and ISH guidelines [7]. In terms of pharmacologic therapy, both the protocols and international guidelines agree that a thiazide diuretic is most cost effective for initial therapy; however, clinicians should also separately consider whether more expensive drugs are indicated for unique patient populations or specific conditions.

While diagnosis and monitoring require only a blood pressure cuff, the treatment of hypertension in MST settings carries additional ethical implications. While some protocols addressed accessible follow-up care as vital in ensuring appropriate dose titration and medication adherence, none discussed the prohibitive costs of renewing medication for many patients in impoverished rural areas, as well as cultural influences and health literacy. Given these concerns, protocols should make clinicians aware of potential discontinuation syndromes (i.e. rebound hypertension or retention of sodium and water), or consequences of misuse and overdose (i.e. hypotension and electrolyte abnormalities) [8,9,10]. Furthermore, NGOs should consider patient education initiatives that emphasize safe medication use, and encourage clinicians to confirm that a patient starting pharmacologic therapy has the financial and logistic means to continue that therapy.

### Diabetes mellitus

Protocols were in agreement with international guidelines for the diagnosis of diabetes, which support the use of point of care glucometers in resource limited settings [11,12], based on a fasting glucose >126 mg/dL (7 mmol/L), >200 mg/dL (11.1 mmol/L) two-hour glucose tolerance, or >200 mg/dL random blood sugar with symptoms [11,12]. No protocol considered diagnosis based on glycosuria with classic diabetes symptoms (sensitivity 21–64%, specificity 98%) [12], which would be a valuable adjunct, considering that urine dipsticks are readily available on most MSTs (Dainton et al., unpublished data).

Although one protocol mentions emergent treatment for hyperglycemia, most do not mention this or other serious indications for urgent referral. These would include biannual review of high-risk patients with previous ulcers, difficulty with foot care, peripheral arterial disease, foot deformities, evidence of neuropathy, with urgent foot care team referral if necessary [12]. Referral is also suggested in the presence of proteinuria with refractory hypertension (greater than 130/80) despite dual pharmacologic therapy [11], or for any blood pressure greater than 160/95 [13]. This lack of such recommendations might result from resource limitations making such referrals unfeasible, or more concerning, due to limited integration of MSTs within the local referral network.

Lifestyle recommendations for weight loss and daily physical activity were largely in line with international guidelines [11,12,14], as were pharmacologic recommendations for metformin and sulfonylurea as first- and second-line therapy respectively [11,12]. Similarly, no protocols mentioned insulin therapy, which is commonly considered either unfeasible [11] or third-line treatment [12] in resource-poor settings. While no protocols specifically mentioned statin therapy, there is also controversy in the literature on its benefit: some suggest treatment for all patients over 40 [11,13], or in high cardiac risk patients like diabetics, even when a lipid profile cannot be measured [12].

For implementation, the protocols we reviewed would require blood glucose strips, a glucose monitor, and urine dipsticks, all of which are readily available on MST brigades (Dainton et al., unpublished data), but which may strain community resources if used for ongoing monitoring or screening [15]. Accordingly, international guidelines suggest that detection programs in resource limited settings should be opportunistic and limited to high risk individuals [12].

There was little mention of monitoring in the protocols. International guidelines suggest that on clinic visits, a random blood sugar <10 mmol/L measured in morning 1.5 to 2 hours after breakfast [16], <6.5 mmol/L fasting or premeal blood sugar, or <9 mmol/L post-meal blood sugar [12] would indicate acceptable control, although self-monitoring remains an alternate option [12]. Furthermore, no protocols discussed other key monitoring parameters, including routine foot examinations for peripheral neuropathy [11,12,13,17], ophthalmologic assessments [11,12,13], measurements of waist circumference, annual urine screening for protein [11,12,13], nor assessment of cardiac risk factors [12]. These monitoring parameters are critical in limiting microvascular and macrovascular morbidity and mortality in diabetic patients.

The absence of any discussion of Type I diabetes in the protocols might arise both from its low prevalence and the limited scope of protocols designed to improve competence in high-yield, common diagnoses. Furthermore, the limited capacity of MST organizations to carry and store perishable medications like insulin in remote locations, the similarly limited capacity of community members to maintain insulin in a usable form, and the high risk of misuse of such medications in low health literacy settings all contribute to an inappropriate environment for management of Type I diabetes on a short-term MST.

### Strengths and Weaknesses

This study responds to evidence of limited use of guidelines by MSTs [4], and makes use of multiple search channels to find unpublished protocols, including Internet and social media. Weaknesses include the low response rate, with a likely bias towards responses from larger, better organized, and better resourced NGOs with greater administrative capacity, as well as those already in possession of clinical protocols. In addition, the initial selection of organizations to be initially contacted was likely biased in favor of those that maintain websites with high search engine optimization and are more likely to be encountered on a Google search. It is also likely that these better-equipped organizations are more likely to possess clinical protocols than their smaller peers.

## Conclusion

Practice guidelines for clinicians treating hypertension and diabetes on MSTs are a key step in the consistent, effective treatment of these two common chronic conditions. This study can be considered an entry point for the creation of harmonized MST guidelines that are acceptable to clinicians, patients, and NGOs. Ideally, consensus guidelines would suggest concise, context-specific case definitions and management options for chronic conditions commonly seen by MSTs in Latin America, and be modified to suit a variety of MST settings and needs.
